# RELi protocol: Optimization for protein extraction from white, brown and beige adipose tissues

**DOI:** 10.1016/j.mex.2019.04.010

**Published:** 2019-04-18

**Authors:** R. Diaz Marin, S. Crespo-Garcia, Ariel Molly Wilson, P. Sapieha

**Affiliations:** a Department of Biochemistry, Maisonneuve-Rosemont Hospital Research Centre, Université de Montréal, Montréal, Québec, H1T2M4, Canada; b Department of Ophthalmology, Maisonneuve-Rosemont Hospital Research Centre, Université de Montréal, Montréal, Québec, H1T2M4, Canada

**Keywords:** Adipose tissue, Protein extraction, Western blot, WAT, BAT

## Abstract

Global obesity rates have reached pandemic proportions, increasing the risk of metabolic complications for hundreds of millions of individuals worldwide. Gaining insight on adipose tissue biology and understanding how fat pads behave during obesity is critical to investigate metabolic syndromes. Elucidation of cellular signaling pathways engaged by adipose tissue both in health and disease requires standardized protocols for protein extraction that yield consistently pure samples. A recurrent problem of currently available protocols is lipid or detergent contamination in extracted protein samples, which renders protein quantification inaccurate and, as a consequence, consistency and reproducibility of protein loading become unreliable. To overcome this problem, we improved the process of adipose tissue protein extraction by improving tissue lysis and decreasing lipid contamination. Here we describe the Removal of Excess Lipids (RELi) protocol to obtain increased yields of total proteins extracted from adipose tissue. The RELi protocol allows accurate and reproducible adipose tissue sample preparation for Western blot analysis and other investigative techniques requiring adipose tissue-derived proteins.

## Graphical abstract

**Figure:**
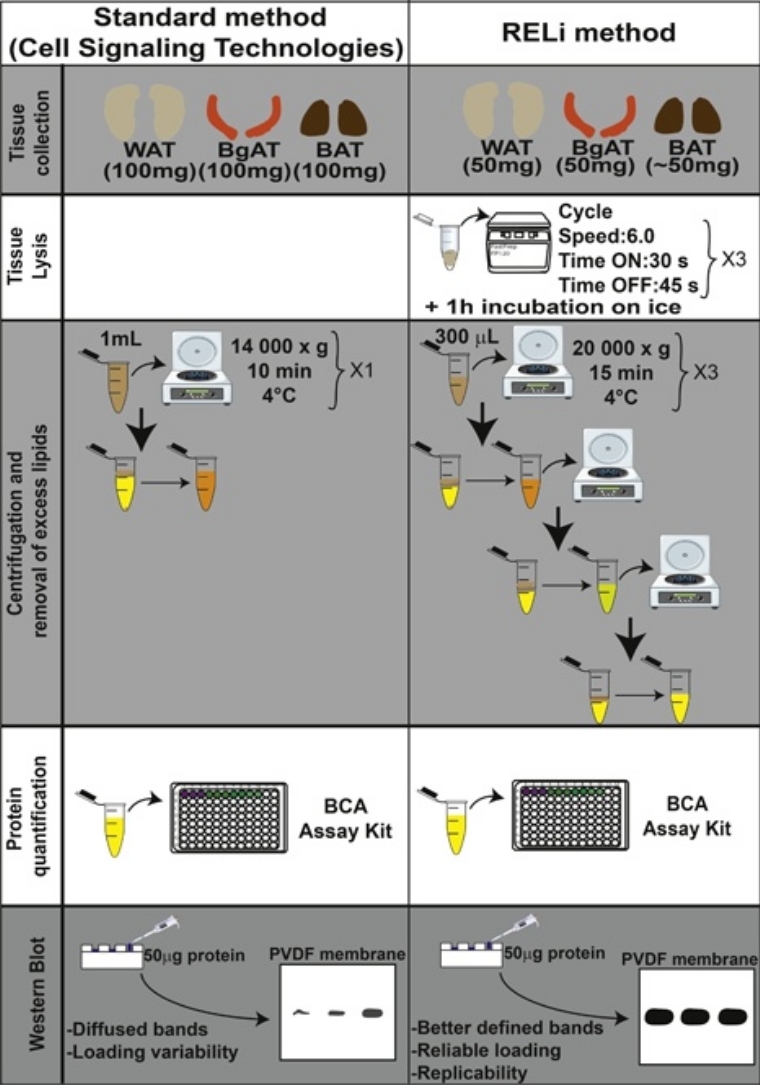

Specifications Table

**Table:** 

Subject Area:	*Biochemistry, Genetics and Molecular Biology*
More specific subject area:	Adipose tissue, protein extraction, Western blot
Protocol name:	Removal of Excess Lipids (RELi) protocol for protein extraction from white, brown and beige adipose tissues.
Reagents/tools:	**Tissue Collection** • Dry ice • Tweezer #5, stainless steel, 11 cm long (Kent scientific corporation, catalog # INS600095) • Dissecting scissors, straight, 10 cm long (Kent Scientific Corporation, catalog # INS600393) • Iris forceps, serrated, 10 cm long (Kent Scientific Corporation, catalog # INS650915) • 60 mm dish (Thermo Scientific, catalog #130181) • 2.0 mL capped tubes (1 per tissue to collect) • Ethanol (EtOH) 70% **Tissue Lysis** • Analytical balance • Fastprep FP120 (SAVANT Instrument Inc) • 1.5 mL capped tubes (4 per tissue to extract) • Ice • Carbon Steel Surgical Blade (Bard-Parker, catalog #311121) • RIPA Buffer (10x) (Cell Signaling Technologies, catalog #9806S) • Distilled water • Protease inhibitor cocktail (Sigma-Aldrich, catalog #P8340) • Phosphatase inhibitor cocktail (Sigma-Aldrich, catalog #P5726) **Centrifugation and removal of excess lipids** • 0.1–10 μl, 2–20 μl, 20–200 μl, and 200–1000 μl micropipettes • 0.1–10 μl, 2–20 μl, 20–200 μl, 200–1000 μl pipette tips • Cooling centrifuge **Protein quantification and sample preparation** • TECAN Infinite M1000 Pro (ThermoFisher Scientific) • Microtest plate 96 well (Sarstedt AG & Co, catalog #82.1581) • BCA kit (Sigma-Aldrich, catalog # QBCA-1KT) • Protein Standard (Sigma-Aldrich, catalog #PO914-10AMP) • Acrylamide gels 12.5% • Laemmli buffer 4X • Molecular weight precision plus protein standards (BIO-RAD, catalog #161-0375) • KimTech Science (Kimberly-Clark Professional) • PVDF membranes for protein blotting (BIO-RAD, catalog #1620177) • Ponceau S solution (Sigma-Aldrich, catalog #P7170-1L) • β -Actin (8H10D10) mouse monoclonal antibody (Cell Signaling Technologies, catalog #3700) • Bcl2 (EPR17509) rabbit monoclonal antibody (Abcam, catalog # ab182858) • eNOS mouse monoclonal antibody (BD Biosciences, catalog # 610296) • Perilipin A rabbit polyclonal antibody (Abcam, catalog # ab3526) • Histone H3 (C-16) goat polyclonal antibody (Santa-Cruz, catalog #sc-8654) • Goat Anti-Mouse IgG (H + L)-HRP Conjugate (BIO-RAD, catalog #1706516) • Goat Anti-Rabbit IgG (H + L)-HRP Conjugate (BIO-RAD, catalog #1721019) • Donkey Anti-Goat IgG (H&L)-HRP (Abcam, catalog #ab97110) • Goat Anti-Mouse IgG (H + L)-HRP Conjugate (BIO-RAD, catalog #1706516) • Clarity Western ECL Substrate (BIO-RAD, catalog #170-5061) • ImageQuant LAS 4000 (GE Healthcare) • ImageJ image processing program (U.S. National Institutes of Health)
Experimental design:	White, brown and beige adipose tissues of C57BL/6 J male mice were collected, and protein was extracted by either the Cell Signaling Technologies (CST) method or by our novel Removal of Excess Lipids (RELi) method. Whole cell lysate proteins were quantified, run by means of Western blot and probed for β-actin levels.
Trial registration:	Not applicable
Ethics:	All experimental procedures were approved by the Animal Care Committee of the Maisonneuve-Rosemont Hospital Research Center and in accordance to the guidelines of the Canadian Council on Animal Care.
Value of the Protocol	• The optimization steps presented in this protocol provide a methodological approach to remove excess contaminating lipids and detergents during protein extraction from adipose tissue. This approach increases the quality and quantity of total extracted proteins from adipose tissue. • This improved protocol permits for proper and reproducible loading of investigated proteins and housekeeping genes in western blot analysis

## Description of protocol

**Fig. 1:**
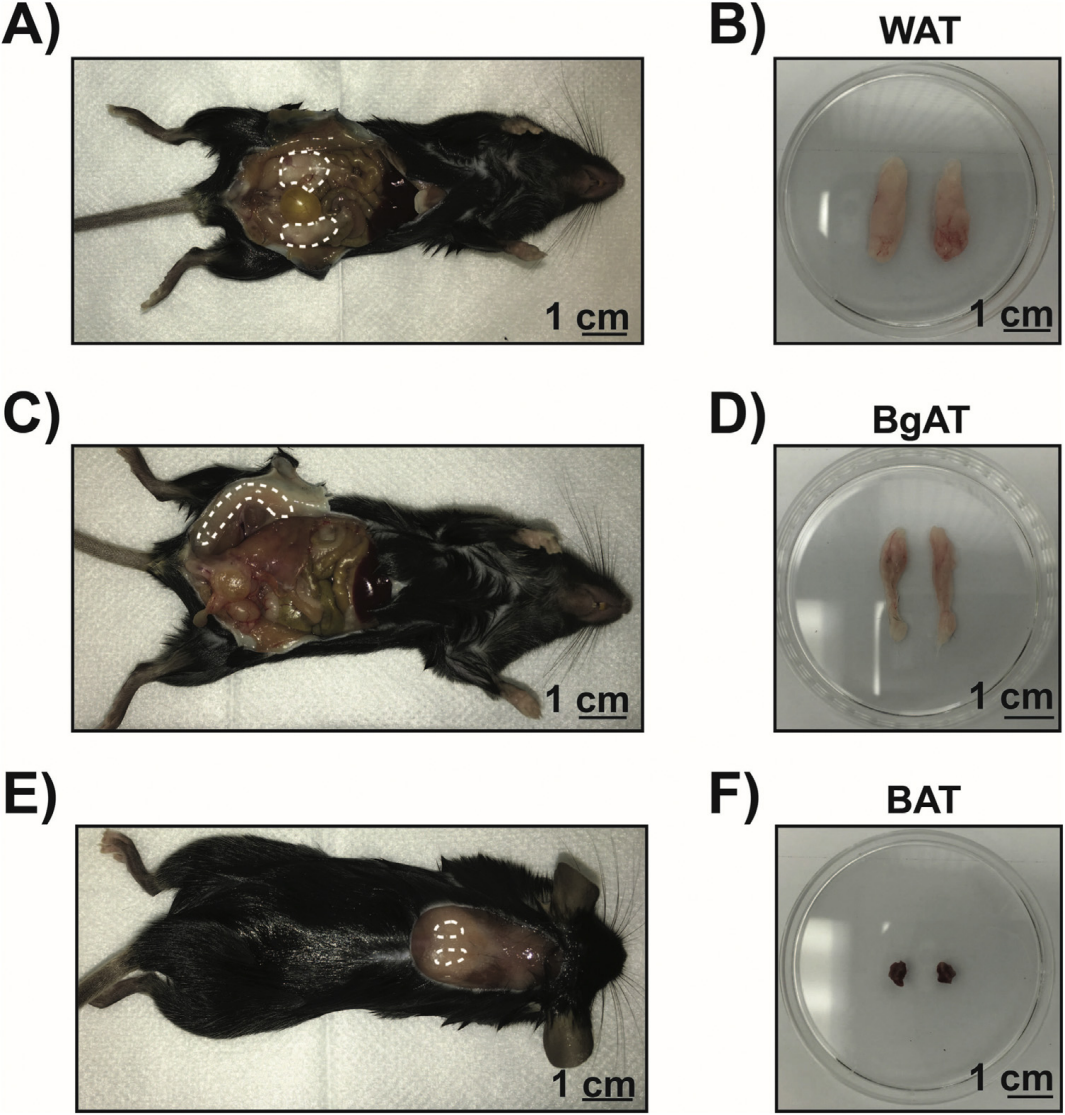
Location of white, brown and beige adipose tissue in mice. A) Representative image of epididymal white adipose tissue (WAT) in male C57BL/6 mice (region delineated by dashed line). B) Epididymal adipose tissue without glands and removed from animals. C) Representative image of interscapular brown adipose tissue (BAT) under the white adipose tissue layer in male C57BL/6 mice (region delineated by dashed line). D) Interscapular adipose tissue removed from animals after extraction of white adipose tissue layer. E) Representative image of subcutaneous beige adipose tissue (BgAT) in male C57BL/6 mice (region delineated by dashed line). F) Subcutaneous BgAT adipose tissue removed from animals.

Adipose tissue (AT) is a major metabolic organ and plays key roles in regulating energy balance. It is highly specialized and distinct fat pads distributed throughout the body have biochemically distinct functions. White adipose tissue (WAT), formed of white adipocytes, plays an important role in energy storage and in the secretion of adipokines, hormones and interleukins [1]. Brown adipose tissue (BAT), formed of brown adipocytes, has the primary function of maintaining core temperature by generating heat through thermogenesis [2]. Within WAT, beige adipocytes are generated as a response to external cues. These adipocytes share similar morphologic features to brown adipocytes, allowing them to participate in thermogenesis [3]. Due to its relevance in physiology and disease, adipose tissue is broadly studied in multiple fields and in multiple experimental models. In mice, epididymal adipose tissue is a classical WAT, interscapular adipose tissue is a classical BAT and the inguinal adipose tissue is beige adipose tissue (BgAT) [4,5] (Fig. 1). There are currently several available approaches to extract protein from adipose tissue such as the bullet blender method or commercially available adipose tissue extraction kits [6,7]. These methods to process adipose tissue, however, require specialized equipment such as a bullet blender or can be expensive when assessing a large number of samples. At present, there is a lack of a standardized protocol for protein extraction from AT for Western blot analysis. Other general protein tissue extraction protocols and kits require limited specific equipment and are affordable but do not factor in the high content of lipids in AT and require high concentrations of detergents that interfere with protein quantification [8,9]. To overcome these problems, we propose a novel method for mouse AT protein extraction based on a commercial RIPA buffer protocol adapted for Western blot protein analysis. The selection of RIPA buffer is based on its versatility for extracting proteins from diverse cell fractions (e.g. membrane, nuclear, cytoplasmic) and its compatibility with follow-up assays. We compared this new Removal of Excess Lipids (RELi) method of protein extraction from AT to the Cell Signaling Technologies method, henceforth referred to as “CST method”. Our optimized protocol provides a simple, affordable and efficient method to extract AT proteins and reduce lipid content. The final outcome is a protocol that is reproducible, provides higher yields and is affordable for AT extraction and subsequent protein analysis.

## Required reagents and equipment

Tissue collection

• Dry ice
• Tweezer #5, stainless steel, 11 cm long (Kent scientific corporation, catalog # INS600095)
• Dissecting scissors, straight, 10 cm long (Kent Scientific Corporation, catalog # INS600393)
• Iris forceps, serrated, 10 cm long (Kent Scientific Corporation, catalog # INS650915
• 60 mm dish (Thermo Scientific, catalog #130181)
• 2.0 mL capped tubes (1 per tissue to collect)
• Ethanol (EtOH) 70%

Tissue lysis

• Analytical balance
• Fastprep FP120 (SAVANT Instrument Inc)
• 1.5 mL capped tubes (4 per tissue to extract)
• Ice
• Carbon Steel Surgical Blade (Bard-Parker, catalog #311121)
• RIPA Buffer (10x) (Cell Signaling Technologies, catalog #9806S)
• Distilled water
• Protease inhibitor cocktail (Sigma-Aldrich, catalog #P8340)
• Phosphatase inhibitor cocktail (Sigma-Aldrich, catalog #P5726)
• Heating thermoblock

Centrifugation and removal of excess lipids

• 0.1–10 μl, 2–20 μl, 20–200 μl, and 200–1000 μl micropipettes
• 0.1–10 μl, 2–20 μl, 20–200 μl, and 200–1000 μl pipette tips
• Cooling centrifuge

Protein quantification and sample preparation

• Plate reader TECAN Infinite M1000 Pro (ThermoFisher Scientific)
• Microtest plate 96 well (Sarstedt AG & Co, catalog #82.1581)
• BCA kit (Sigma-Aldrich, catalog # QBCA-1KT)
• Protein Standard (Sigma-Aldrich, catalog #PO914-10AMP)
• 12.5% acrylamide gels
• Laemmli buffer 4X
• Molecular weight precision plus protein standards (BIO-RAD, catalog #161-0375)
• SimplyBlue SafeStain (Invitrogen, catalog #LC6060)
• KimTech Science (Kimberly-Clark Professional)
• PVDF membranes for protein blotting (BIO-RAD, catalog #1620177)
• Ponceau S solution (Sigma-Aldrich, catalog#P7170-1 L)
• β-Actin (8H10D10) mouse monoclonal antibody (Cell Signaling Technologies, catalog #3700)
• Bcl2 (EPR17509) rabbit monoclonal antibody (Abcam, catalog # ab182858)
• eNOS mouse monoclonal antibody (BD Biosciences, catalog # 610296)
• Perilipin A rabbit polyclonal antibody (Abcam, catalog # ab3526)
• Histone H3 (C-16) goat polyclonal antibody (Santa-Cruz, catalog #sc-8654)
• Goat Anti-Mouse IgG (H + L)-HRP Conjugate (BIO-RAD, catalog #1706516)
• Goat Anti-Rabbit IgG (H + L)-HRP Conjugate (BIO-RAD, catalog #1721019)
• Donkey Anti-Goat IgG (H&L)-HRP (Abcam, catalog #ab97110)
• Goat Anti-Mouse IgG (H + L)-HRP Conjugate (BIO-RAD, catalog #1706516)
• Clarity Western ECL Substrate (BIO-RAD, catalog #170-5061)
• ImageQuant LAS 4000 (GE Healthcare)
• ImageJ image processing program (U.S. National Institutes of Health)

## Protocol

Tissue collection

1 Anesthetize mice with 3% isoflurane (or other method of anesthesia).
2 Perform a cervical dislocation and proceed to adipose tissue collection.
3 White adipose tissue (WAT) collection:

3.1 Spray EtOH 70% on the abdomen of the mouse.

3.2 Make an incision with 10cm long dissecting scissors and cut longitudinally to expose the peritoneum.

3.3 The localization of mouse WAT is shown in Fig. 1A (delineated by a dashed line).

3.4 After extracting the WAT from the abdominal cavity, using scissors, gently remove the secretory glands attached to the adipose tissue in the region closest to the testicles, as shown in Fig. 1B.

4 Beige adipose tissue (BgAT) collection:

4.1 After collecting WAT, proceed as indicated above (steps 3.1–3.4).

4.2 With tweezers, pull the skin away from the peritoneum to expose the subcutaneous space where the beige adipose tissue is found, as indicated in Fig. 1C.

4.3 The location of BgAT in mice is depicted in the Fig. 1C (delineated by dashed line).

4.4 With 10cm long dissecting scissors, carefully remove BgAT (Fig. 1D).

5 Brown adipose tissue (BAT) collection:

5.1 Spray EtOH 70% on the back of the mouse.

5.2 Make a longitudinal incision of approximately 2 cm at the level of the shoulders.

5.3 BAT is located under a layer of WAT as shown in Fig. 1E (location of BAT is located within the dashed line, under the visible WAT).

5.4 With tweezers, carefully grab the furthest right-hand part of the white adipose tissue layer and cut with scissors, from right to left, removing both white and brown fat pads from the scapula.

**Fig. 2:**
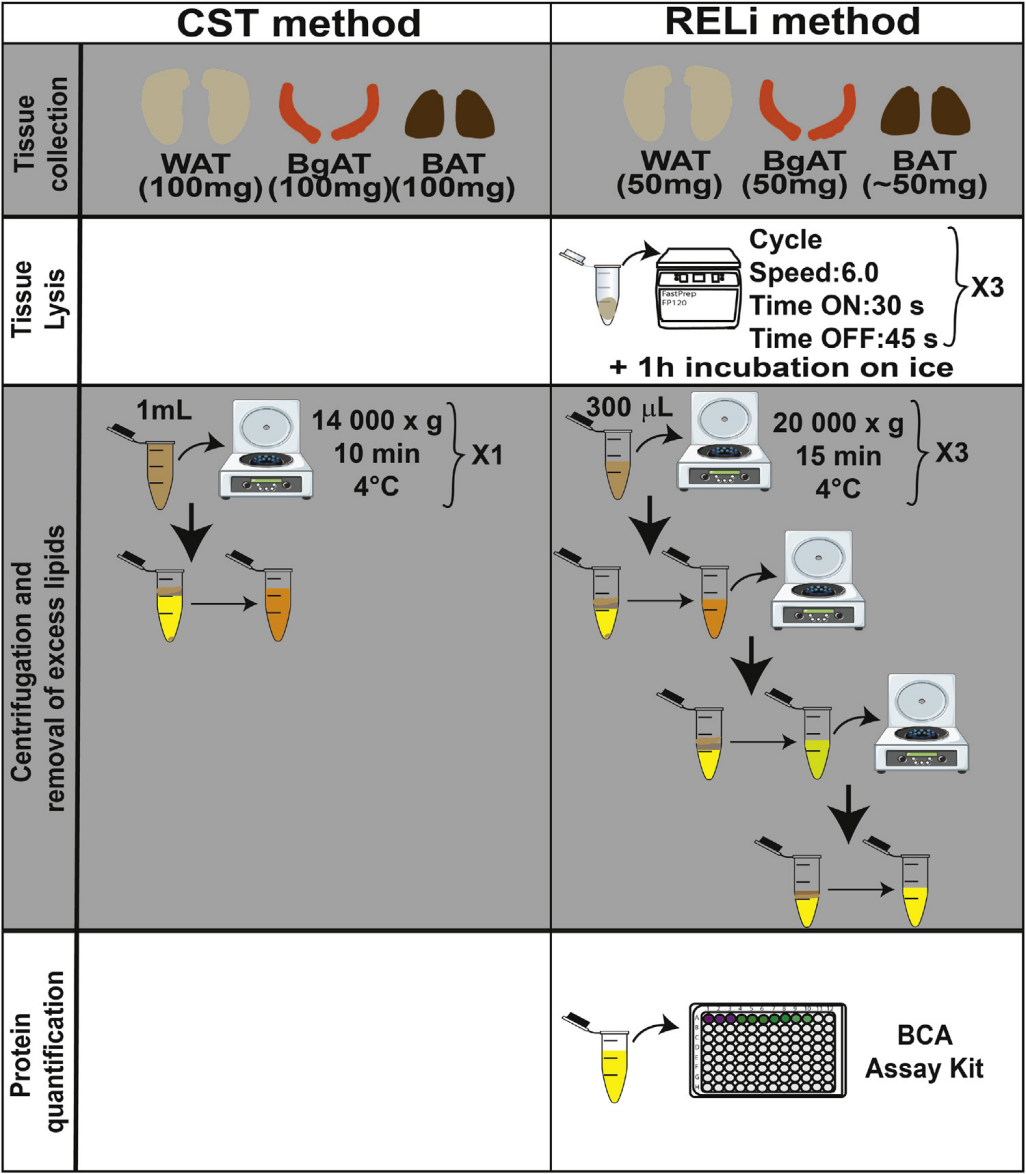
RELi protein extraction method compared to the old standard method. The schematic illustrates the different steps of protein extraction using the CST and RELi methods applied to distinct adipose tissues: I) Tissue collection, II) Tissue lysis, III) Centrifugation and removal of excess lipids, and IV) Protein quantification.

5.5 Place the excised chunk of tissue (white adipose tissue layer containing the brown adipose tissue pads) in a dissection dish and remove all white adipose tissue (BAT has a darker color than WAT), as depicted in Figs. 1F and 2 .

6 Place the adipose tissues in a 1.5mL capped tube and keep in dry ice.

### Tissue lysis

7 Weight a 1.5 mL capped tube and tare the balance.
8 Cut the adipose tissue while it is still frozen and add 100 mg to the 1.5 mL capped tube.

Note: keep the tube containing the adipose tissue in dry ice until ready to extract all other samples.

9 Prepare necessary volume of RIPA 1X solution (300μL/50mg of adipose tissue are needed per sample) supplemented with protease and phosphatase inhibitor cocktails for a final concentration of 1/500 and 1/100 respectively.
10 Add 300μL of RIPA 1X supplemented with protease and phosphatase inhibitors to the 1.5mL capped tubes and keep on ice.
11 Set the Fastprep FP120 machine’s speed to 6.0 m/s and lyse tissue for 30 s.
12 Remove samples from the Fastprep FP120 machine and chill samples on ice for 45 s.
13 Repeat steps 11–12 two more times or until tissue is completely lysed.
14 Incubate samples on ice for at least 1h.
15 If any solid portion of the adipose tissue remains in the 1.5 mL capped tube, remove it with the help of forceps before continuing onto the next steps.

Centrifugation and removal of excess lipids

16 Centrifuge samples at 4°C, 20 000×*g* for 15 min.
17 Remove the layer of lipids (top layer) from the 1.5 mL capped tube and transfer ˜ 200 μL of supernatant to a new 1.5 mL capped tube.

Note: It is very important to collect the smallest amount of fat possible when transferring the supernatant as this will affect protein purity and quality.

18 Repeat steps 16–17 at least 2 more times to reduce the amount of lipids in the samples.

Protein quantification and sample preparation

19 Prepare a standard curve for the protein quantification with bovine serum albumin (BSA) in a 96-well plate or alternative method for protein quantification.
20 Prepare dilutions 1/100 for BAT and 1/20 for WAT and BgAT for every sample. Add the diluted samples to the same 96-well plate.
21 Measure absorbance of the dilutions at 562 nm with the BCA kit using a plate reader.
22 Calculate the volume of protein needed to load 50μg of protein for Western blot, add Laemmli buffer and complete to a total volume of 30μL with RIPA buffer 1X or distilled water.
23 Boil samples for 5 min in a heat block at 100ºC.

Extraction validation by Western blot

24 Load 15μL of the protein preparation for every sample on a 12.5% acrylamide gel and migrate at 120 V for 1h.

Note: After migration, the gel can be colored with SimplyBlue solution to verify protein loading. Wash the gel 3 times for 5 min with Milli-Q water to remove SDS and buffer salts. Color the gel with SimplyBlue solution for 2h at RT. To obtain a clear background, decolor gel with Milli-Q water during 1h at RT and place a KimTech on top of the gel to absorb the dye. Take a picture of the gel with a digital imaging system (ImageQuant) and quantify total protein loaded per well with Image J.

25 Transfer proteins on a PVDF membrane for 1h 30min at 100V, 4 ºC.
26 Note: After transfer, Ponceau S solution can be used to verify protein loading. Wash the membrane twice with TBS + Tween (TBST), for 5 min to remove all Ponceau off the membrane prior to continuing analysis. Block the membrane with 5% non-fat milk diluted in TBST, for 1h at room temperature (RT).
27 Incubate O/N 4ºC with primary antibody or 1h at RT for β-actin primary antibody.
28 Wash membrane 3 times with TBST for 7 min.
29 Incubate the membrane with secondary antibody for 1h at RT.
30 Wash the membrane 3 times with TBST for 7 min.
31 Develop membrane with a digital imaging system (ImageQuant) using the Clarity Western ECL Substrate.
32 Quantify mean intensity of the bands detected with Image J or alternative gel quantification system.

## Method validation

The validation of the above-described method was obtained by means of Western blot analysis comparing the RELi protocol to the CST method. WAT, BAT and BgAT were collected from three adult male C57BL/6 mice and proteins were extracted using both methods with RIPA buffer. Protein concentration was evaluated with the BCA method by measuring absorbance (562 nm) with a TECAN infinite M1000 Pro (Table 1). Samples processed with the CST methods show overestimated protein concentrations due to interference from lipid contamination when compared to our optimized protocol.

**Table 1:** Protein concentration using the CST and RELi methods.

CST Method				
Adipose tissue type	Final concentration (mg/mL)	Total Volume (mL)	Total concentration (mg)	Initial amount of tissue (mg)
WAT 1	8.8210944	0.4	3.528438	100
WAT 2	8.1973348		3.278934	
WAT 3	6.1786221		2.471449	
BAT 1	12.237029		4.894811	
BAT 2	20.062376		8.02495	
BAT 3	17.000284		6.800113	
BgAT 1	2.5041111		1.001644	
BgAT 2	8.2426992		3.29708	
BgAT 3	6.7683584		2.707343	

RELi Method				
Adipose tissue type	Final concentration (mg/mL)	Total Volume (mL)	Total concentration (mg)	Initial amount of tissue (mg)
WAT 1	7.8344202	0.16	1.253507	50
WAT 2	7.7210094		1.235361	
WAT 3	6.9498157		1.111971	
BAT 1	17.1704		2.747264	
BAT 2	11.613269		1.858123	
BAT 3	10.876099		1.740176	
BgAT 1	7.2787071		1.164593	
BgAT 2	9.0025517		1.440408	
BgAT 3	8.8097533		1.409561	

**Fig. 3:**
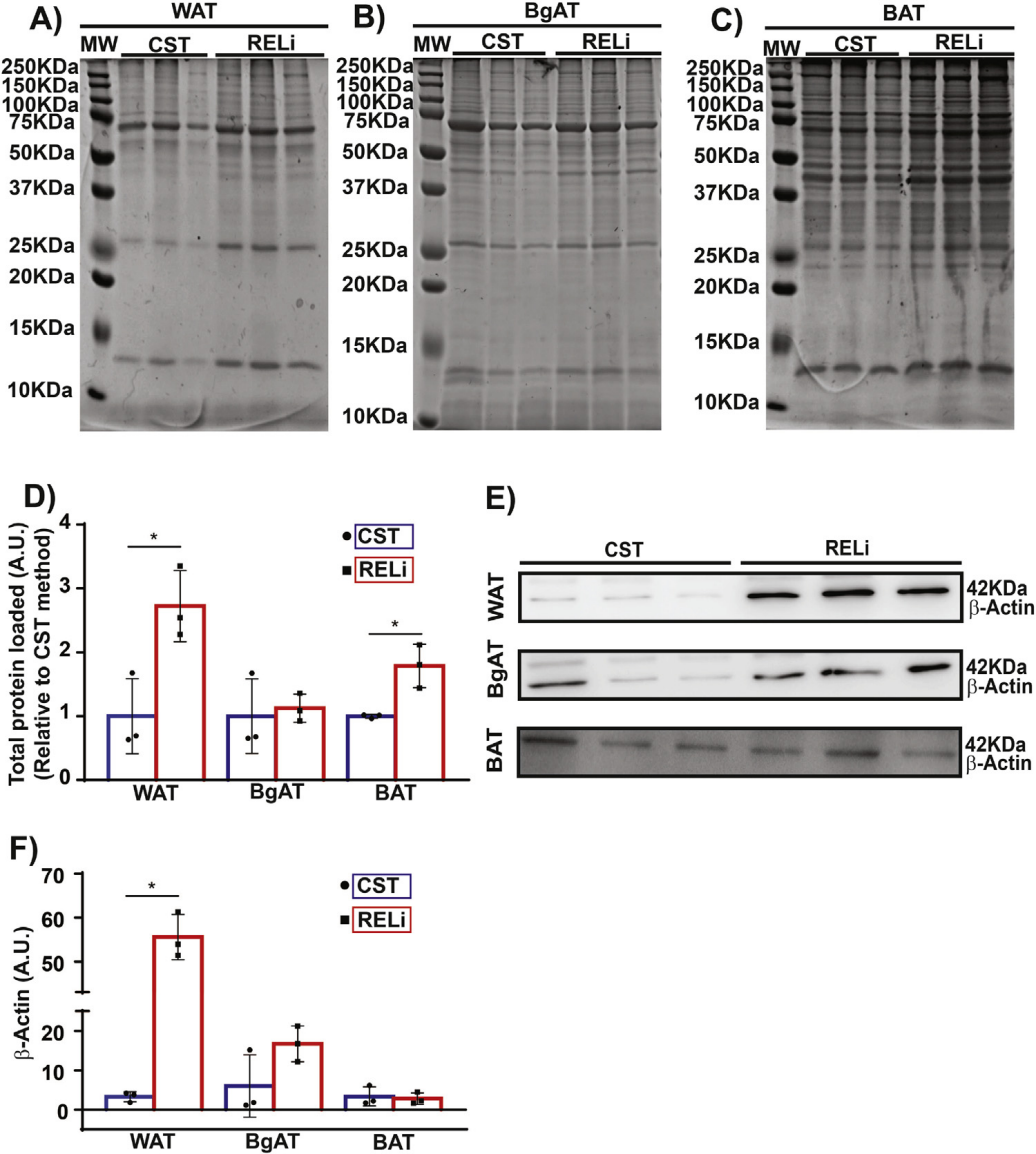
Assessment of protein extraction efficiency with the CST and RELi methods. Western blot analysis was performed on proteins extracted from 3 independent samples following the CST and RELi method. A–C) SimplyBlue SafeStaining stained gel showing protein extracts from WAT (A), BgAT (B) and BAT (C). MW, protein molecular weight standard; CST, CST method and RELi, Removal of Excess Lipids method. D) Quantification of total protein loaded for WAT, BAT and BgAT following SimplyBlue SafeStaining. E) Western blot analysis of β-actin (˜42 KDa) in WAT, BgAT and BAT extracts. F) Quantification of total protein loaded for WAT, BAT and BgAT. Data shown as mean ± S.E.M. of triplicate wells and are representative of two independent experiments; *p < 0.05, Student’s unpaired *t*-test.

To validate the interference of lipids in protein quantification, 50 μg of protein were loaded for every tissue sample on 12.5% acrylamide gels, separated by SDS-PAGE electrophoresis and counterstained with SimplyBlue SafeStain. Considering that all lanes were loaded with the same amount of calculated protein, samples processed with our RELi method presented a higher and more consistent amount of proteins when compared to that obtained from the CST method for all analyzed tissues (WAT, BAT and BgAT) (Fig. 3A–D).

To assess the outcome of removal of excess lipids during protein extraction, we performed Western blot analysis on the samples extracted with the CST and RELi method for all three adipose tissues. β-actin was used as a housekeeping protein to assess the high variability in the loading. Our results indicate that the CST method yielded inferior amounts of protein from WAT and BgAT (Fig. 3E–F) as determined by β-actin expression when compared to our optimized protocol. The amount of β-actin detected in BAT, however, was not improved by our method. This is probably due to the lower levels of lipids in BAT compared to other types of adipose tissue.

**Fig. 4:**
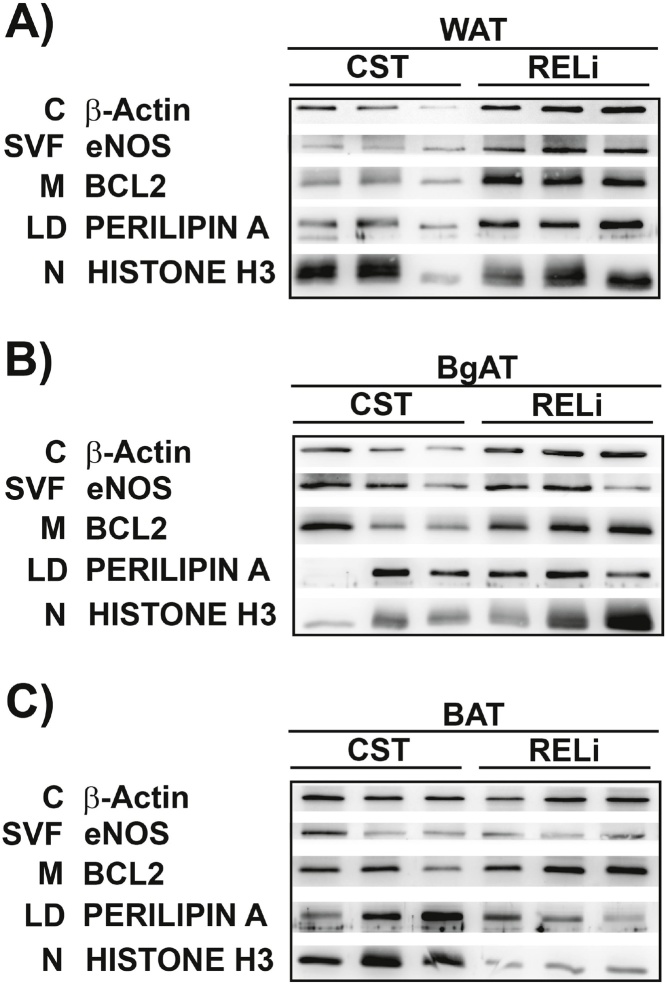
Assessment of quality and uniformity of adipose tissue proteins extracted with the CST and RELi methods. A–C) Western blot analysis of adipose tissue cellular compartments: Cytosol (β-actin ˜42 KDa), Stromal vascular fraction (eNOS ˜140 KDa), Mitochondria (BCL2 ˜26 KDa), Lipid associated proteins (Perilipin A ˜68 KDa), Nuclear protein (Histone H3 ˜15 KDa) in WAT, BgAT and BAT extracts; C (Cytosol), SVF (Stromal vascular fraction), M (Mitochondria), LD (Lipid droplet associated), N (Nuclear).

To validate the quality and uniformity of adipose tissue proteins, we evaluated intracellular compartments in adipose tissue by Western blot. Markers of cellular fractions included: cytosolic (β-actin), stromal vascular fraction (eNOS), mitochondrial (BCL2), lipid droplet (LD) associated proteins (Perilipin A) and nuclear (Histone H3) for all three types of adipose tissue [10]. Our results show that the RELi method yields higher levels of intracellular fractions when compared to the CST method for WAT and certain fractions of BgAT such as cytosol (Fig. 4A–B) as determined by the enhanced expression of the above-mentioned markers. Conversely, for BAT, the RELi method did not show superiority (Fig. 4C). This may be due to initially lower volumes used for tissue lysis. Nevertheless, when compared to other protocols that use TCA precipitation to extract adipose tissue proteins, our RELi method remains a quicker and more reliable technique for adipose tissue extraction for Western blot analysis [10]. A consistent quantification of protein after extraction is fundamental for reproducibility between samples and thus our optimized protocol will help researchers obtain more reliable protein analysis data.

## Sources of funding

RDM is supported by a research scholarship from Faculté des Études Supérieures et Postdoctorales de l’Université de Montréal. SCG is supported by a research grant from Fonds de Recherche Santé Québec. This work was supported by operating grants to P.S. from the Canadian Institutes of Health Research (353770), the Canadian Diabetes Association (OG-3-14−4544-PS), the Heart & Stroke Foundation Canada (G-16-00014658), The Foundation Fighting Blindness Canada, and Natural Sciences and Engineering Research Council of Canada (418637). P.S. holds the Wolfe Professorship in Translational Research and a Canada Research Chair in Retinal Cell Biology.

## Disclosures

The authors declare no conflicts of interest. All experimental procedures were approved by the Animal Care Committee of the Maisonneuve-Rosemont Hospital Research Center and in accordance to the guidelines of the Canadian Council on Animal Care.
